# Increased Loading, Efficacy and Sustained Release of Silibinin, a Poorly Soluble Drug Using Hydrophobically-Modified Chitosan Nanoparticles for Enhanced Delivery of Anticancer Drug Delivery Systems

**DOI:** 10.3390/nano7110379

**Published:** 2017-11-08

**Authors:** Cha Yee Kuen, Sharida Fakurazi, Siti Sarah Othman, Mas Jaffri Masarudin

**Affiliations:** 1 Department of Cell and Molecular Biology, Faculty of Biotechnology and Biomolecular Sciences, Universiti Putra Malaysia, 43400 Serdang, Selangor, Malaysia; yeekuen_91@hotmail.com (C.Y.K.); sarahothman@upm.edu.my (S.S.O.); 2 Cancer Research Laboratory, Institute of Biosciences, Universiti Putra Malaysia, 43400 Serdang, Selangor, Malaysia; 3 Department of Human Anatomy, Faculty of Medicine and Health Sciences, Universiti Putra Malaysia, 43400 Serdang, Selangor, Malaysia

**Keywords:** chitosan nanoparticles, hydrophobically-modified CNP, slibinin, nanobiotechnology

## Abstract

Conventional delivery of anticancer drugs is less effective due to pharmacological drawbacks such as lack of aqueous solubility and poor cellular accumulation. This study reports the increased drug loading, therapeutic delivery, and cellular accumulation of silibinin (SLB), a poorly water-soluble phenolic compound using a hydrophobically-modified chitosan nanoparticle (pCNP) system. In this study, chitosan nanoparticles were hydrophobically-modified to confer a palmitoyl group as confirmed by 2,4,6-Trinitrobenzenesulfonic acid (TNBS) assay. Physicochemical features of the nanoparticles were studied using the TNBS assay, and Attenuated total reflectance Fourier transform infrared spectroscopy (ATR-FTIR) analyses. The FTIR profile and electron microscopy correlated the successful formation of pCNP and pCNP-SLB as nano-sized particles, while Dynamic Light Scattering (DLS) and Field Emission-Scanning Electron Microscopy (FESEM) results exhibited an expansion in size between pCNP and pCNP-SLB to accommodate the drug within its particle core. To evaluate the cytotoxicity of the nanoparticles, a Methylthiazolyldiphenyl-tetrazolium bromide (MTT) cytotoxicity assay was subsequently performed using the A549 lung cancer cell line. Cytotoxicity assays exhibited an enhanced efficacy of SLB when delivered by CNP and pCNP. Interestingly, controlled release delivery of SLB was achieved using the pCNP-SLB system, conferring higher cytotoxic effects and lower IC_50_ values in 72-h treatments compared to CNP-SLB, which was attributed to the hydrophobic modification of the CNP system.

## 1. Introduction

Over the last decade, radiation, chemotherapy and surgery have become important methods to treat cancer patients. Among these methods, chemotherapy remains the most significant treatment, with synthetic drugs being the most potent chemotherapeutic drugs used [1]. The main obstacle faced by current treatments is the inefficient delivery of drugs to target cells. Thus, conventional treatment often leads to side effects in patients due to non-specific interaction of the drugs with non-cancerous cells in the body. Silibinin, also known as silybin, is a major active constituent obtained from the seeds of the milk thistle plant, *Silybum marianum*. It exists as a polyphenolic flavonolignan with hepatoprotective and antioxidant activity. Substantial evidence from current research has demonstrated its clinical use in medical therapies especially in Asia, Europe, and the USA [2,3,4]. It has been suggested that silibinin is an extremely utilizable natural compound for cancer studies [5,6]. Additionally, there have been increasing research endeavors conducted to elucidate the potential anticancer properties of silibinin against numerous cancer models, including lung [7,8], skin [9,10], prostate [11,12], colon [13], and bladder [14]. Silibinin is preferentially advantageous for development as a candidate therapeutic agent as it is considered remarkably safe in cytotoxicity studies, as it confers low toxicity even in acute administration dosages in both humans and animals, and there are no LD_50_ values for the flavonolignan reported in laboratory studies. However, its widespread application in medical therapy has been marred by its low bioavailability due to low solubility in water (0.4 mg/mL) [15,16,17,18]. This restriction was due to its multiple ring structure, which is too large to be absorbed by simple diffusion, which leads to poor water solubility, poor bioavailability and thus poor intestinal absorption [19].

In order to circumvent such a disadvantage, numerous methods for its synthesis have been proposed in order to increase the solubility and bioavailability of silibinin. This has included the implementation of solid lipid nanoparticles via encapsulation [20], incorporation in solid dispersion systems [21], and complexation with phospholipids [22]. A previous study by Zhang et al. utilized stearic acid as a colloidal carrier for the encapsulation of silibinin. This modification successfully incorporated silibinin into solid lipid nanoparticles with anticipated shape, size and entrapment efficiency. However, the systemic adoption of solid lipid nanoparticles for the encapsulation of silibinin is currently limited to the inherent rapid in vivo clearance by the reticuloendothelial system (RES) which decreases the persistence of the silibinin-nanoparticle complex from blood circulation. Considering the nano-scale dimensions of nanoparticles, they can be adapted to utilize the enhanced permeability and retention effects (EPR) of tumor vasculatures to increase the accumulation of chemotherapeutic drugs. Nanoparticles have been demonstrated to exhibit 200 times higher drug accumulation in tumor cells compared to normal tissues, such as kidney, muscle and heart [23].

Chitosan (CS) is a linear polysaccharide linked by a β-(1-4)-linked-d-glucosamine backbone with randomly acetylated amine groups. It is a product of the partial deacetylation of chitin and can be acquired from numerous sources including the exoskeleton of crustaceans [24]. CS is known for its superior features such as biocompatibility, biodegradable and non-toxicity, which makes it an excellent candidate to be utilized in agriculture, biomedicine, food industries and many other fields. A previous study by Kang et al. [25] has successfully synthesized chitosan nanoparticles (CNP) loaded with albendazole, a poorly water-soluble but extremely permeable anthelmintic drug, where a stable nanoparticle system was established. Besides this, Chai et al. [26] also demonstrated that CNP can function as a drug delivery system for Doxorubicin, an anticancer drug. The study showed that Doxorubicin was successfully coated by chitosan and alginate with a good combination and showed controlled release properties. Thus, CNP can be hydrophobically-modified to incur hydrophobic–hydrophobic interactions with drugs to encapsulate them into the nanoparticles, which can potentially increase the solubility of low bioavailability drugs, and protect and stabilize the drugs from the surrounding environment.

This study demonstrates the enhanced encapsulation efficiency of silibinin in hydrophobically-modified chitosan nanoparticles as compared with traditional chitosan nanoparticles, in order to enhance the therapeutic efficacy in the A549 human lung cancer cell line. Based on the background and objectives, perhaps novel, effective and safe hydrophobically-modified chitosan nanoparticles for the enhanced therapeutic delivery of silibinin is proposed. Findings from this study can perhaps be used to further increase the efficacy of silibinin in anti-cancer applications.

## 2. Results and Discussion

### 2.1. Particle Size and Polydispersity of Nanoparticle Samples

**Figure 1:**
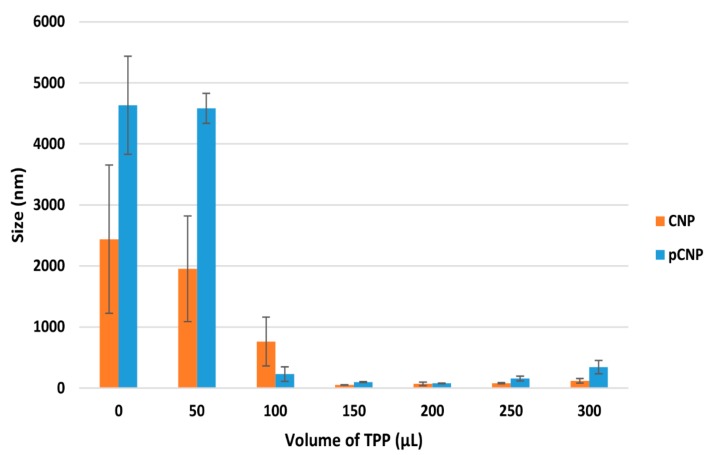
The PSD of nanoparticles at different TPP volumes. The smallest size of nanoparticles was obtained with the addition of 150 μL TPP for CNP and 200 μL for pCNP. Error bars represent the SEM averaged from three independent replicates of the experiment. The one-way analysis of variance (ANOVA) was performed with a *p* value of the particle size at 0.0002 and <0.0001 for CNP and pCNP, indicating that there was a significant effect of TPP volume on the particle size formed.

**Figure 2:**
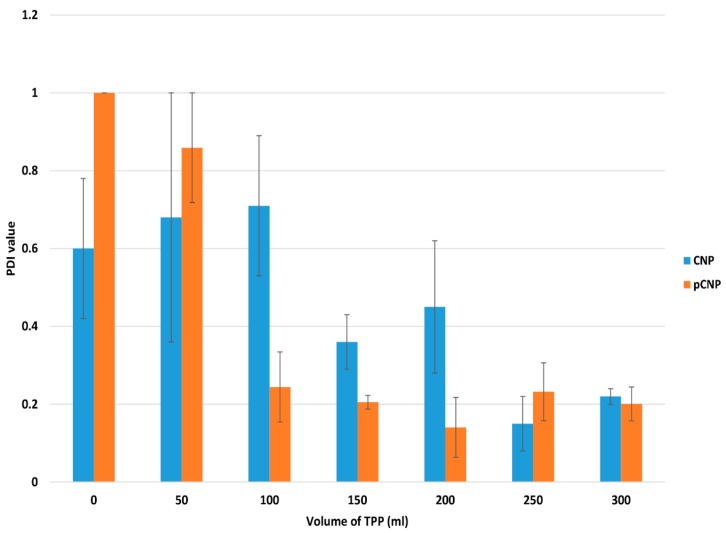
PDI of nanoparticles at different TPP volumes. The lowest PDI of nanoparticles was obtained with the addition of 250 μL TPP for CNP and 200 μL for pCNP. Error bars represent the SEM averaged from three independent replicates of the experiment. The one-way analysis of variance (ANOVA) was performed with *p* value of particle size <0.0001 for both CNP and pCNP indicating that there was a significant effect of TPP volume on the PDI of the nanoparticles formed.

The formation of CNP occurred through ionic crosslinking between protonated amine groups of CS and anionic phosphate groups of the cross linker, Sodium Tripolyphosphate (TPP) [27,28]. When cross linking occurs, the protonated amine groups will associate with the anionic phosphate groups of TPP to form nanoparticles where the CS polymer will start to form spherical, nano-scaled particles. Figure 1 and Figure 2 depict the particle size distribution (PSD) and polydispersity index (PDI) of nanoparticles formed by the crosslinking of 600 μL CS with different volumes of TPP. The graph suggested that the formation of nanoparticles occurred at the minimal addition of 100 μL TPP for pCNP and 150 μL for CNP. Figure 1 and Figure 2 show where the size of the pCNP experienced a noticeable drop from 4583.00 ± 244.62 nm to 228.11 ± 119.06 nm, while the PDI dropped from 0.577 nm to 0.113 nm with the addition of 50 μL and 100 μL of TPP; the size of the CNP decreased from 761.40 ± 399.73 nm to 49.14 ± 3.98 nm, while the PDI decreased from 0.71 ± 0.18 nm to 0.36 ± 0.07 nm with the addition of 100 μL and 150 μL of TPP. This observation was similar to the earlier study of Masarudin et al. (2015), where a 50 μL addition of TPP was suggested as the minimal volume required to form CNP.

The smallest size of nanoparticles was obtained using 150 μL of TPP for CNP (49.14 ± 3.98 nm) and 200 μL for pCNP (77.08 ± 6.50 nm), respectively. The lowest PDI of nanoparticles was obtained using 250 μL of TPP for CNP (0.15 ± 0.07) and 200 μL for pCNP (0.14 ± 0.08), respectively. The PDI was used as an indication of nanoparticle homogeneity, which depicts the stability of the samples in the colloidal suspension [29]. The PDI showed a similar trend as PSD, where the value dropped at the TPP ranges of 50–150 μL. Both the PSD and PDI data suggested that a decrease in nanoparticle size occurred with an increase of the cross linker, while in turn increasing the monodispersity of the nanoparticles up to 200 μL of TPP addition. This trend was most probably contributed to by the increased volumes of TPP that provided more anionic phosphate groups to form electrostatic interactions with the protonated CS chain [30]. As more TPP was added, the cross-linking degree of the nanoparticles was augmented to where a compact particle structure is thought to occur, leading to the production of smaller-sized nanoparticles [31].

Interestingly, when the TPP volume exceeded 200 μL, both the particle size and PDI increased to above 100 nm and 0.2, respectively. It possibly indicates that agglomeration between the nanoparticles occurred. The addition of anionic TPP beyond the optimum volume will attract more CS chains due to the increase in the degree of cross-linking, and will possibly attract the neighboring nanoparticles causing agglomeration [32], which suggests that 200 μL of TPP and 600 μL of pCS can form nanoparticles of optimum size and PDI.

### 2.2. Utilization of the Free Amine Group by Nanoparticles Formation

**Figure 3:**
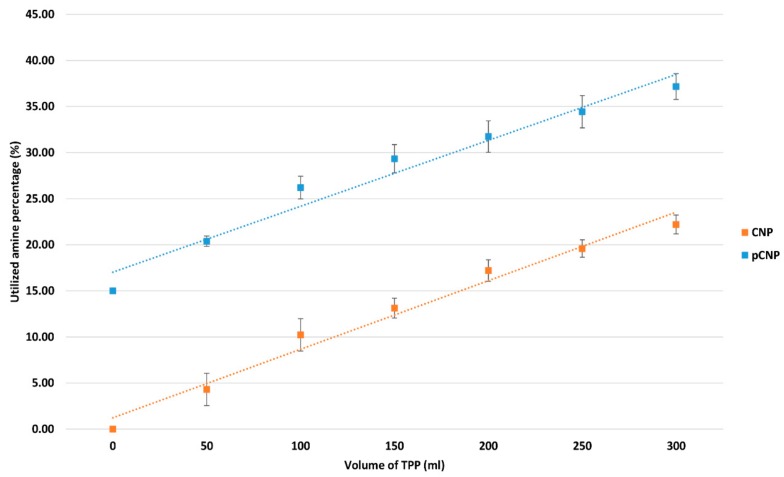
Utilized amine percentage at different volumes of TPP. The utilized amine percentage increased with the addition of TPP volume for both CNP and pCNP. The graph data are presented as mean ± SEM from three independent experiments. A paired *t*-test was performed with a *p* value below 0.0001, which indicates that CNP and pCNP have extremely significant differences on the percentage of free NH_2_ group.

The formation of CNP and pCNP was suggested to be due to the ionic crosslinking interactions between protonated free amine group of CS/pCS and the anionic phosphate group of TPP. When the amount of cross-linker added was increased, the amount of amine groups being utilized will also increase, which in turn decreases the amount of free amine group available in a constant volume of CS/pCS. Figure 3 shows that the percentage of utilized amine groups of chitosan increased in direct proportion to the increased volume of TPP added to the CS/pCS, from 0 up to 22.20 ± 1.02 and 37.17 ± 1.397 for CNP and pCNP, respectively, and upon addition of 300 μL of TPP. The data suggested that cross-linking occurred between CS/PCS and TPP on the formation of CNP and pCNP. This increases postulates that CNP and pCNP were successfully constructed, which is similar to the observation reported by a previous study [29]. A higher percentage of utilized amine groups was detected in the pCNP compared to CNP, probably due to the interaction of palmitic acid with the CS prior to the TPP. The palmitic acid will conjugate to the amine group of the CS to form pCS, where utilization of the amine group occurs, and is then only further cross-linked with TPP to form pCNP, which explains the higher percentage of utilization compared to CNP.

### 2.3. Formation of pCNP and pCNP-SLB

The encapsulation efficiency (% E.E) of pCNP were approximately 49.33 ± 1.45%, which suggests that when approximately 50% of the SLB had been loaded into the pCNP, there was about a 2.5-fold increase of the particle size increment. Anyway, the % E.E of CNP were approximately only 24.58 ± 2.06%, which suggests that only half of the SLB will be loaded into the CNP compared to the pCNP. A higher % E.E was achieved by the pCNP compared with the CNP, probably due to the hydrophobic anchor possessed by the palmitic acid that conjugated with the CS, forming a hydrophobic core, where the SLB will associate with the core instead of the hydrophilic surface. It suggested that the pCNP system is a great carrier candidate that can increase the loading of hydrophobic load. The study of Marchiori et al. [33], performed nanoencapsulation by using interfacial deposition of the preformed polymer method with pomegranate oil as an oil core for the encapsulation of SLB. In their study, a high % E.E was achieved, credited to the higher affinity of SLB with the oil core than the aqueous phase. These results was similar to the findings of our study, where higher % E.E was obtained in pCNP compared to CNP, as the SLB is suggested to have associated with the pCNP by hydrophobic–hydrophobic interaction.

**Figure 4:**
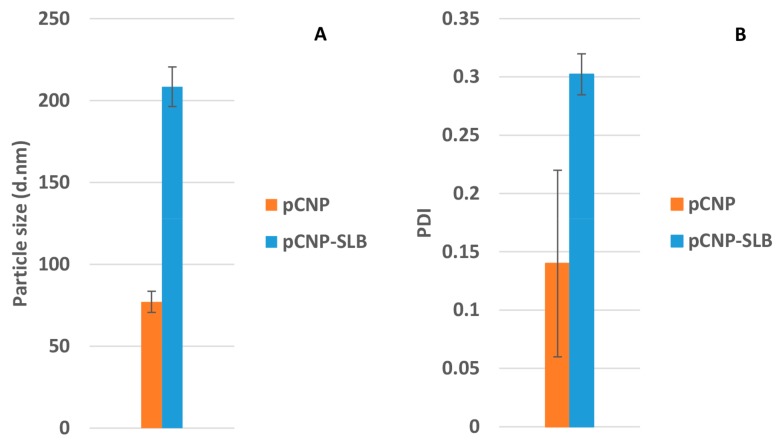
(**A**) PSD and (**B**) PDI of pCNP after SLB encapsulation. The particle size and PDI of pCNP increased after the encapsulation of SLB. The graph data is presented as mean ± SEM from three independent experiments. A *p* value below 0.05 was obtained, indicating significant differences in particle size between pCNP and pCNP-SLB as measured by paired *t*-test.

On the other hand, the study of Shankar and Argawal successfully encapsulated SLB by using polymeric micelles. Polymeric micelles are self-assembled, amphiphilic copolymers where the hydrophilic part usually forms the shell and hydrophobic part forms the core, which is similar to the structure of pCNP. The study also achieved a high % E.E, suggesting that amphiphilic nanoparticles are a great candidate for hydrophobic SLB [34]. The size and PDI of the pNCP-SLB are shown in Figure 4. It is important to optimally obtain small-size nanoparticles as well as encapsulated nanoparticles, because the nano-scaled particles are able to extravasate through the endothelium in the epithelium, inflammatory sites, and tumors, or penetrate the microcapillaries. This possesses an advantageous effect in which the nanoparticles permit efficient uptake by many different cell types in addition to selective drug accumulation at target sites [35].

Particle size date showed an expansion of about 270%, from 77.08 ± 6.50 nm to 208.43 ± 12.02 nm, upon SLB loading (300 μM). Similarly, the PDI of the pCNP increased from 0.14 ± 0.08 to 0.30 ± 0.02 after encapsulation, suggesting the successful encapsulation of SLB in pCNP. The expansion in size indicated the successful incorporation of SLB within the core of the nanoparticles. The slight changes in PDI occurred due to the decrease of the uniformity in the size of the nanoparticles following encapsulation, which consist of a population of free pCNPs of smaller size (<100 nm) and pCNP-SLBs of larger size (>100 nm). The expansion in size was correlated with the encapsulation efficiency (% E.E) of the nanoparticles shown in Table 1.

### 2.4. Morphological Analysis of Nanoparticles by FESEM 

**Figure 5:**
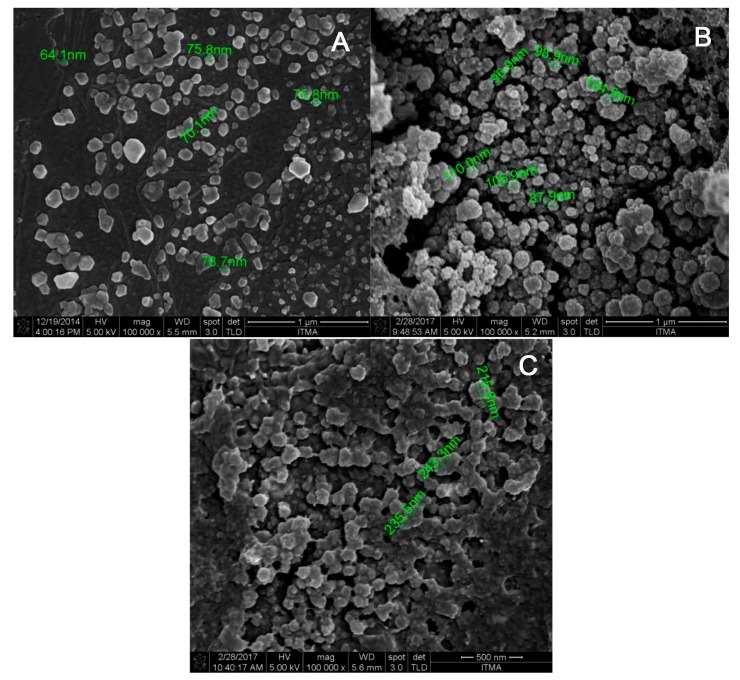
The figure shows the morphological structure of (**A**) CNP, (**B**) pCNP and (**C**) pCNP-SLB. The CNP shows a particle size of 64.1 nm to 84.7 nm, the pCNP shows a particle size of 87.9 nm to 110.0 nm, and the pCNP-SLB shows a particle size ranging from 215.8 nm to 243.3 nm.

A morphological analysis was performed using FESEM to ascertain the structure, size and polydispersity of CNP, pCNP and pCNP-SLB. As shown in Figure 5, the nanoparticles are generally spherical in shape, with a size ranging from below 100 nm before encapsulation and up to 200–300 nm post-encapsulation. As described in the study of Ariff et al., the CS or pCS in this study appeared to have an irregular shape when they stood alone, and to form spherically-shaped nanoparticles with the addition of TPP, indicating the success of the CNP/pCNP formation [36]. Blank CNP particles conferred sizes in the range of 64.1 nm to 84.7 nm, whilst pCNP was slightly larger at 87.9 nm to 110.0 nm diameter.

When SLB was encapsulated into the nanoparticles, the expansion in size detected from the DLS was made morphologically evident using FESEM. The pCNP-SLB samples existed as larger particles with sizes approximately 215.8 nm to 243.3 nm. It was postulated that the increase in size of pCNP was attributed to the incorporation of palmitic acid within its core, which led to a significant expansion after the encapsulation of SLB in pCNP. This result was similar to the results observed previous by Ariff et al., where the size of nanoparticles expanded post-encapsulation [34]. A previous study by Leena et al. [18], utilized chitosan nanoparticles loaded with different concentrations of SLB and incorporated alginate/gelatin scaffolds for bone formation in vitro. The particle size of CNP-SLB obtained from the study was about 264 ± 51 nm post-encapsulation with 100 μM of SLB. Regarding the pCNP-SLB in this study, where an initial SLB concentration of 300 μM was used for encapsulation, a smaller size of nanoparticles with a higher % E.E was obtained in our study. This further suggests that pCNP is a better nano-carrier for SLB. Anyway, some agglomeration was observed in Figure 5, which can possibly be attributed to the drying process used while preparing the samples for FESEM imaging [37].

### 2.5. Identification of Characteristic Functional Groups in Nanoparticle Samples

**Figure 6:**
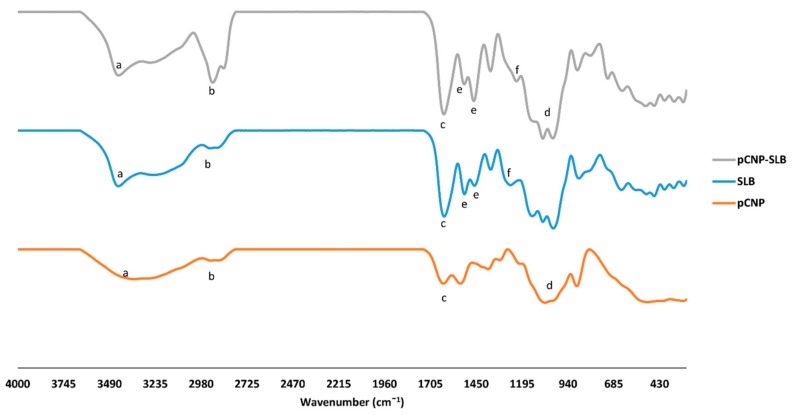
FTIR spectra of pCNP, SLB and pCNP-SLB. The characteristic functional groups are labeled for (**a**) amine group, (**b**) alkyl group, (**c**) carbonyl group, (**d**) inorganic phosphate group, (**e**) aromatic ring and (**f**) ether group.

In order to study the formation of drug-encapsulated pCNP-SLB, an FTIR analysis was performed to study the presence of important functional groups belonging to SLB and pCNP that occurred in pCNP-SLB. According to Coates [38] every single molecule has a unique vibrational spectrum which can be utilized as a fingerprint to be compared between an “unknown” and a “known” spectra. Thus, by comparing the peaks of the “known” spectra of SLB and pCNP with the “unknown” spectra of pCNP-SLB, we can deduce the formation of pCNP-SLB. The infrared spectra for SLB, pCNP, and pCNP-SLB is as shown in Figure 6, while a summary of the important characteristic functional groups detected is depicted in Table 2.

There are several characteristic peaks shown by pCNP: a broad peak at 3353 cm^−1^, corresponding to the hydrogen-bonded O–H stretching vibration and N–H stretching from the primary amine overlapped in the same region; a peak at 1632 cm^−1^, corresponding to the amide II carbonyl stretch; and a peak at 1067 cm^−1^, corresponding to the inorganic phosphate group of TPP [39]. Besides this, several characteristic peaks are also possessed by SLB: at 3446 cm^−1^, due to –OH stretching; at 2936 cm^−1^, due to –CH stretching; at 1626 cm^−1^, due to –C=O stretching; at 1507 and 1461 cm^−1^, due to –C=C skeleton vibrations of aromatic ring stretching; and at 1268 cm^−1^, due to –C–O–C stretching. From the table, the percentage of transmittance at the peak of 3353 cm^−1^ of pCNP and SLB was 49.62% and 49.98%, respectively, but this increased to 85.97% after encapsulation, indicating a decrease in the primary amine group. It shows that upon encapsulation, the free primary amine group was utilized and led to the shift in the peak from 3353 cm^−1^ in pCNP to 3416 cm^−1^ in pCNP-SLB. A similar observation was shown by the study of Kafshgari et al. [40] where the amine group of CS was shifted from the wavenumber of 3413 cm^−1^ to 3395 cm^−1^ upon encapsulation of bovine serum albumin due to the utilization of amine groups.

However, the inorganic phosphate group of pCNP detected at wavenumber of 1067 cm^−1^ and 1081 cm^−1^ for pCNP-SLB, with increased transmittance after encapsulation, indicated the utilization of the functional group. The characteristic peak for alkyl groups at 2888 cm^−1^ and 2936 cm^−1^ was detected in pCNP and SLB, respectively. After encapsulation, this peak appeared at 2851 cm^−1^, with a decrease of transmittance, indicating that more active alkyl groups are present upon encapsulation, possibly due to the introduction of SLB in the pCNP [41]. Meanwhile, the peaks attributed to carbonyl groups of pCNP and SLB were detected at the 1632 cm^−1^ and 1626 cm^−1^ wavenumber, respectively, and shifted to 1635 cm^−1^ after encapsulation, also with an increase of transmittance, which shows the interaction between pCNP and SLB that utilized the C=O bond; these results were similar to the previous study of Tan et al. [42]. The C–O–C stretching that happened only in SLB was also observed in pCNP-SLB, with an increase in transmittance from 10 to 67.95%, indicating the utilization of the functional group after encapsulation.

### 2.6. CNP and pCNP Cytotoxicity in A549 Cell Lines

CNP is generally considered as non-cytotoxic to cells, due to its proven biodegradable and biocompatible properties [43]. In order to assess the effectiveness of its hydrophobically-modified counterpart, pCNP as a safe carrier for SLB delivery, an MTT cytotoxicity assay was performed in the A549 cell line against the nanoparticle system. MTT is used to assess initial cytotoxicity because we need to ensure that that vectors itself do not exert cytotoxicity effects. After incubation, those metabolically-active viable cells will produce purple formazon salts and can be read at 570 nm by using a spectrophotometer as an indicator for viable cells [44].

**Figure 7:**
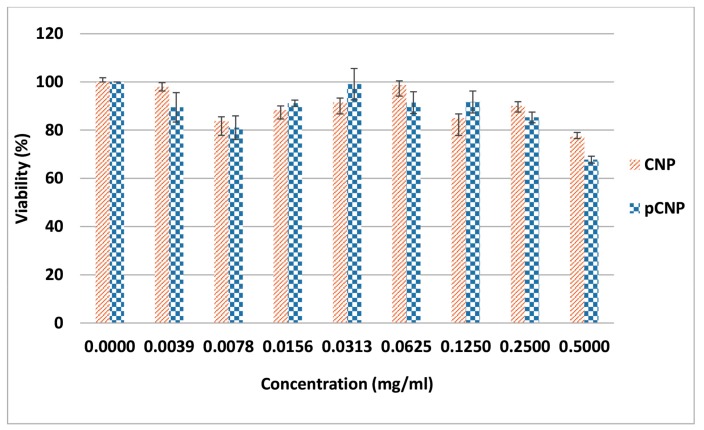
The percentage of viability assessed by CNP and pCNP using an MTT assay. CNP and pCNP have similar cytotoxicity effects where no IC_50_ was found. Data are presented as mean ± SEM from three independent experiments. One-way ANOVA was performed with *p* = 0.1097, which indicated that both CNP and pCNP had no significant difference in cytotoxicity effect in A549 cell lines. The experiment was not conducted with a further time point study because it showed no cytotoxicity effects in the 24 h study.

Figure 7 shows the viability of A549 cells after being treated by CNP and pCNP to compare the cytotoxicity of pCNP after hydrophobic modification, and the results demonstrate a similar cytotoxicity effect by both carriers where no IC_50_ was found. As mentioned in the previous study of Masarudin [45] there is no significant deviation between CNP and pCNP in term of size, morphology and also content. Thus, we also assumed that there is no substantial difference in terms of the cytotoxicity effect, and this was postulated by the results. It is important to verify the non-toxic effects of pCNP so that the delivery efficacy of SLB will not be affected by the vector in the later assay. The previous study of Zhang et al. [46] utilized hydrophobically-modified oleoyl-chitosan (OCH) as carriers for doxorubicin, and the vector did not confer cytotoxicity effects to A549 cell lines, which is similar to our results. Besides this, the study of Chiu et al. [47], also revealed that N-palmitoyl chitosan nanoparticles with different degrees of substitution had no significant cytotoxicity effect in HT1080 cells, which further indicated that pCNP is a good candidate for a delivery vector.

### 2.7. Nanoparticle-Mediated Cell Cytotoxicity of SLB CNP-SLB and pCNP-SLB in A549 Cell Lines

**Figure 8:**
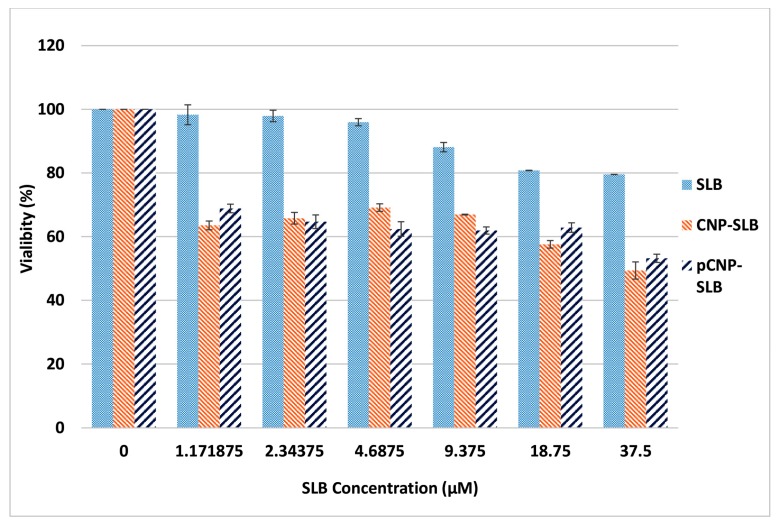
Cytotoxic effect of SLB, CNP-SLB and pCNP-SLB after 48 h of treatment. A similar SLB efficacy for CNP-SLB and pCNP-SLB was observed where both are significantly higher than SLB alone. Data are presented as mean ± SEM from three independent experiments. One-way ANOVA was performed with a *p* value below 0.05 for both between SLB and CNP-SLB and between SLB and pCNP-SLB, which indicated a significant difference of efficacy between SLB alone and SLB delivered by vector, and *p* = 0.9995 between CNP-SLB and pCNP-SLB, which indicated no significant difference of efficacy between them.

The MTT assay was repeated to assess the efficacy of SLB in A549 cells using CNP-SLB and pCNP-SLB systems to discern whether a nanoparticle-mediated delivery system can augment its in vitro efficiency. Approximately 300 μM of SLB was used for the encapsulation of CNP and pCNP to form CNP-SLB and pCNP-SLB, as suggested by the study of Mateen et al. [48], where 10–75 μM of SLB was able to inhibit the cell growth of non-small cell lung cancer (NSCLC) [49]. Based on the E.E data obtained from Table 1, both CNP-SLB and pCNP-SLB contained approximately 75 μM (24.57% E.E) and 150 μM (49.34% E.E) of SLB respectively, and the final concentration for cell treatment was 37.5 μM. Figure 8 shows the percentage of cell viability for cells treated with SLB, CNP-SLB, and pCNP-SLB, which generally revealed a substantial improvement in efficacy for SLB delivered using CNP and pCNP 48 h post treatment. Since the concentration of SLB encapsulated in pCNP was two-fold greater compared to CNP, it was initially thought that a lower IC50 would be obtained 48 h post-treatment. The IC_50_ calculated was 18.36 μM and 18.69 μM for CNP-SLB and pCNP-SLB, respectively. At this time point, the difference is thought to be less significant between CNP and its hydrophobically-modified version.

**Figure 9:**
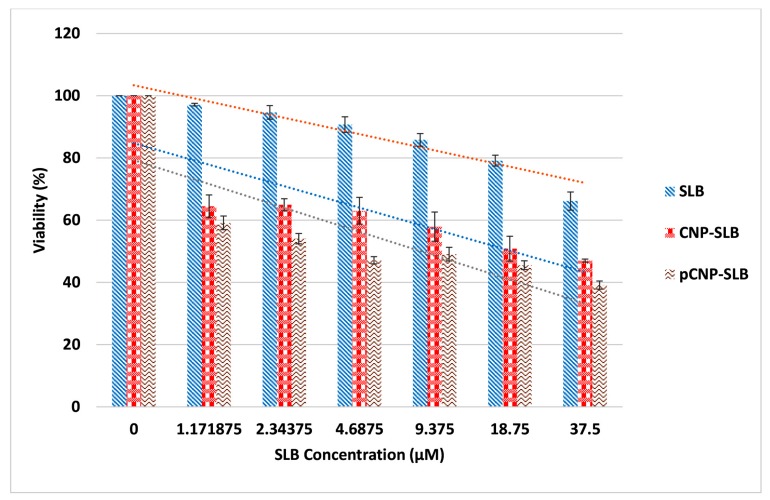
Cytotoxic effect of SLB, CNP-SLB and pCNP-SLB after 72 h of treatment. The highest efficacy was shown by pCNP-SLB, followed by CNP-SLB and SLB alone. Data are presented as mean ± SEM from three independent experiments. A one-way ANOVA was performed and a *p* value below 0.05 indicated a significant difference of efficacy between them.

Interestingly, after 72 h of treatment, the IC_50_ for pCNP-SLB decreased to 6.058 μM, and that of CNP-SLB decreased to 12.77 μM, which is about 211% difference, as shown in Figure 9. This change was postulated to demonstrate the effectiveness of using pCNP as a controlled-release carrier, where the SLB was shown to be released slowly from the nanoparticle core upon treatment. Consequently, the efficacy of SLB for the first 48 h post-treatment was similar for both CNP and pCNP systems, but significantly differed for treatment of 72 h. This was most probably due to the fact that most of the SLB encapsulated in CNP was fully released at 48 h post-treatment, compared to pCNP, due to a greater association of SLB to the hydrophobic core in pCNP. This suggests that pCNP is great candidate for controlled-release properties, coupled with a higher % E.E for SLB. A previous study by Mateen et al. [48] demonstrated that an SLB treatment of 10–75 μM will cause growth inhibition in large cell carcinoma cells (H1299 and H460) and a bronchioalveolar carcinoma cell line (H322), which is similar to the findings of our study. However, the results of SLB delivery was improved by using pCNP, which shown by the low IC_50_.

The incorporation of palmitic acid into CNP formed pCNP, which provides a hydrophobic anchor to associate with SLB through a hydrophobic-hydrophobic interaction. As proposed by Masarudin [45], a higher drug efficacy was obtained when [^14^C]-doxorubicin was delivered using pCNP compared to CNP as a carrier, which is comparable with the results in this study. The pCNP was able to achieve a higher % E.E when more SLB was encapsulated into the hydrophobic core, attributed to the hydrophobic-hydrophobic interaction and then delivered and released in a slower manner for longer duration compared to CNP. This explained why the efficacy of SLB for the first 48 h post treatment was similar, but differed considerably for the treatment of 72 h.

## 3. Materials and Methods 

Chitosan (CS, low molecular weight), sodium tripolyphosphate (TPP), palmitic acid N-hydroxy-succinimide ester (NHS-palmitate), silibinin and dimethyl sulfoxide (DMSO) were acquired in powder form from Sigma-Aldrich (St. Louis, MO, USA). Roswell Park Memorial Institute-1640 medium (RPMI-1640), fetal bovine serum (FBS), 0.25% trypsin-EDTA (1×) and Antibiotic-Antimycotic (100×) were purchased from Gibco Life Technologies (Grand island, NY, USA). Glacial acetic acid, sodium hydroxide and hydrochloric acid (analytical grade) were obtained from Friendemann Schmidt Chemicals (Parkwood, Western Australia). Absolute ethanol was purchased from Nacalai Tesque, Inc. (Kyoto, Japan). All reagents, unless otherwise stated, were used without further purification.

### 3.1. Synthesis of Chitosan Nanoparticles (CNP) and Palmitoyl Chitosan Nanoparticles (pCNP)

#### 3.1.1. Chitosan Nanoparticles (CNP)

CNPs were synthesized by ionic gelation methods as described by Masarudin et al. (2015) [29]. CS and TPP were prepared to a concentration of 1.0 mg/mL in 50 mL centrifuge tubes before subsequently diluted to 0.5 mg/mL and 0.7 mg/mL respectively, and adjusted to pH 5 and pH 2 using 1 M NaOH and 1 M HCl. The CNPs were formed by adding increasing volumes of TPP solution (0 μL to 300 μL) to 600 μL of CS solution. The CNPs were then purified by centrifugation at 13,000 rpm for 20 min. After centrifugation, 40% of the total CNP supernatant volume were mixed with 60% of deionized water (dH_2_O) corresponding to the 40% supernatant volume and utilized for further analyses.

#### 3.1.2. Hydrophobically-Modified Chitosan Nanoparticles (pCNP)

The 1.0 mg/mL CS was prepared as mentioned above and adjusted to pH 6. Separately, NHS-palmitate was prepared in absolute ethanol to a concentration of 0.9 mg/mL. NHS-palmitate was then added to the CS solution through dropwise addition at a 2:1 volume ratio, before being left to react for 20 h at 50 °C. Following incubation, the hydrophobically-modified chitosan (pCS) was precipitated from the solution by adjusting the pH to pH 9. The precipitate was subsequently separated from the solution by centrifugation at 4000 rpm for 45 min. The precipitate was washed once with a solution of acetone: ethanol (50:50) and then thrice with dH_2_O, and left to dry in the oven. The pCS was then used to prepare pCNP using similar methods as previously described for CNP.

#### 3.1.3. Synthesis of Silibinin-Encapsulated pCNP (pCNP-SLB)

5 mg of Silibinin was dissolved in 50% DMSO and 50% dH_2_O to prepare a 10 mM master stock. It was then further diluted by mixing 100 μL of master stock with 100 μL of dH_2_O until it became 5 mM and was added dropwise to 600 μL of pCS with continuous mixing until a final concentration of 300 μM was then added with 200 μL of TPP.

### 3.2. Physicochemical Characterization of Nanoparticle Samples

#### 3.2.1. Analysis of Particle Size Distribution and Polydispersity 

The size and distribution of synthesized CNPs, pCNPs, CNP-SLB and pCNP-SLB were ascertained using dynamic light scattering on a Malvern Zetasizer Nano S Instrument (Malvern Instruments, Worcestershire, UK). About 1000 μL of samples was loaded into a disposable cuvette, and the particle size was measured in triplicate readings for each sample to ensure the stability of the sample. All data were recorded as mean ± standard error of mean (SEM). The significant alteration of particle sizes between the CNP and pCNP samples was subsequently evaluated by one way analysis of variance (ANOVA), with a *p* value < 0.05 considered as significant.

#### 3.2.2. Morphological and Surface Topological Analyses

The surface morphologies and size of pCNP and pCNP-SLB were further examined using field-emission scanning electron microscopy (FESEM) analysis. Nanoparticles were diluted prior to analysis by mixing 100 μL of samples with 500 μL of dH_2_O. A drop of each diluted samples was then placed onto an aluminum stub and left to dry in an oven for three days. The sample-loaded stubs were then gold-coated in a vacuum before being observed under an electron microscope (FEI NOVA nanoSEM 230, Thermo Fisher, Hillsboro, OR, USA).

#### 3.2.3. Fourier Transform Infrared (FTIR) Analysis with Attenuated Total Reflectance (ATR-FTIR)

FTIR analysis was performed for SLB, pCNP and pCNP-SLB samples. Prior to analysis, the samples were freeze dried using a Coolsafe 95-15 PRO freeze drier (ScanVac, Lynge, Denmark) for 48 h. Next, the samples in powder form was analyzed using a Spectrum 100 (Perkin Elmer, Waltham, MA, USA) using the attenuated total reflectance (ATR) method at an infrared frequency range of 200 to 4000 cm^−1^.

### 3.3. Determination of Free Amine Groups in Nanoparticle Samples Using the 2,4,6-Trinitrobenzene Sulfonic Assay

Prior to the assay, 0.05% (*v*/*v*) TNBS reagent, 0.1 M NaHCO_3_, 10% (*w*/*v*) SDS and 1.0 M HCl were prepared in a 15 mL centrifuge tube. CS standards were prepared by serially diluting 50 μL of 0.5 mg/mL CS solution using 0.1 M NaHCO_3_. Approximately 50 μL of 0.05% (*v*/*v*) TNBS solution was then added to each sample in Eppendorf tubes. pCS standards were subsequently prepared using similar methods as for CS. Next, 100 μL of nanoparticle samples at different TPP volume additions were aliquoted into Eppendorf tubes and mixed with 100 μL 0.05% (*v*/*v*) TNBS solution. All the standard and sample tubes were then incubated for 3 h at 37 °C. Following incubation, 100 μL of the standard/samples were transferred into a 96-well plate. Approximately 100 μL of 10% SDS and 75 μL of 1 M HCl were added into each well and mixed, and the absorbance was read at a wavelength of 405 nm. The utilized amine percentage was then calculated using the following equation:

(1) $$\mathrm{Free}\;\mathrm{amine}\;\mathrm{percentage}\;\left(\%\right)\;=\frac{A_{405}\;\mathrm{of}\;\mathrm{CNP}\;/\mathrm{pCNP}}{\begin{array}{c} A_{405}\;\mathrm{of}\;\mathrm{CS}/\mathrm{pCS}\\ \left(\mathrm{at}\;\mathrm{same}\;\mathrm{concentration}\;\mathrm{used}\right)\; \end{array}}\times \;100\%$$

The significant alteration of utilized amine percentage of CNP and pCNP samples was subsequently evaluated by paired *t*-test, with a *p* value < 0.05 considered as significant.

### 3.4. Encapsulation Efficiency of Silibinin in Nanoparticle Samples

The encapsulation efficiency (% EE) was calculated by comparing the difference in absorbance at 288 nm between free SLB and the supernatant of encapsulated SLB. The CNP-SLB and pCNP-SLB samples were centrifuged at 13,000 rpm for 20 min. The supernatant was then collected and the absorbance at 288 nm was read using a UV/VIS spectrophotometer (NP80, Implen, München, Germany). The % EE was then calculated using the following equation:

(2) $$\%\;\mathrm{EE}\;=\;\frac{{\;A}_{288}\mathrm{of}\;\mathrm{free}\;\mathrm{SLB}-A_{288}\;\mathrm{of}\;\mathrm{SLB}\;\mathrm{in}\;\mathrm{supernatant}}{A_{288}\;\mathrm{of}\;\mathrm{free}\;\mathrm{SLB}\;}\;\times 100\%$$

The absorbance of free SLB at 288 nm was used to deduct the absorbance of SLB that remained in the supernatant post-encapsulation and divided by the absorbance of free SLB, then multiplied by 100 percent to get the percentage of encapsulation (% E.E).

### 3.5. Determination of In Vitro Cellular Efficacy Using Nanoparticle-Mediated Delivery of Silibinin 

The A549 cell line was established by culturing in a T-25 flask with growth media consisting of 90% of 1× RPMI medium 1640 and 10% FBS. Approximately 100 μL of cells were seeded into a 96-wells plate. Cells were seeded at a density of 7 × 10^3^ cells per well for 48-h treatments and 4 × 10^3^ for 72-h treatment, whilst cells were treated with 100 μL of CNP, pCNP, SLB, CNP-SLB and pCNP-SLB. At the end of each time point, the wells were decanted and replaced with 170 μL fresh growth media. Separately, 5 mg/mL MTT solution was prepared in 1× PBS and sterilely filtered with 0.22 μm nylon filter. Approximately 30 μL of the MTT solution was then added into each well, mixed and incubated for a further 4 h. Following incubation, 150 μL of solution was removed and 100 μL of DMSO was added to each well. The absorbance was then read at 570 nm using a Bio-Rad iMark™ Microplate Absorbance Reader (Bio-Rad, Hercules, CA, USA). Cell viability was determined using the following equation:

(3) $$\;\%\;\mathrm{viability}=\;\frac{A_{570}\;\mathrm{of}\;\mathrm{treated}\;\mathrm{cells}}{A_{570}\;\mathrm{of}\;\mathrm{untreated}\;\mathrm{cells}\;}\;\times 100\%$$

The significant efficacy of nanoparticles, SLB and SLB-encapsulated nanoparticles was subsequently evaluated by one way analysis of variance (ANOVA), with a *p* value < 0.05 considered as significant.

## 4. Conclusions

The present study showed the successful hydrophobic-modification of CNP and encapsulation of SLB. In addition, pCNP was found to be a better delivery vector compared to CNP due to the incorporation of NHS-palmitic acid, which is non-toxic and possesses a hydrophobic-hydrophobic interaction with SLB, achieving a higher encapsulation efficiency and higher efficacy effect in the A549 cell line compared to CNP. Hence, the pCNP system established in this study shows great potential for imminent customization and studies as a delivery vector, especially for low bioavailability drugs for cancer therapeutics.

## Figures and Tables

**Table 1:** % E.E of CNP-SLB and pCNP-SLB at 300 μM SLB. The results show that the % E.E of pCNP-SLB is about two-fold higher that CNP-SLB. The graph data are presented as mean ± SEM from three independent experiments.

Sample	% E.E CNP-SLB	% E.E pCNP-SLB
1	22.46	50.12
2	28.70	51.35
3	22.58	46.51
Average	24.58 ± 2.06	49.33 ± 1.45

**Table 2:** The functional groups present in pCNP, SLB and pCNP-SLB. All the characteristic functional groups of pCNP, SLB and pCNP-SLB are listed in the table at their respective wavenumber and percentage of transmittance.

Functional Group	Wavelength	Percentage Transmittance	Sample
Amine (a) (N–H)	3353	49.62	pCNP
	3446	49.98	SLB
	3416	85.97	pCNP-SLB
Alkyl group (b) (–CH, CH_2_)	2888	80.94	pCNP
	2936	88.91	SLB
	2851	24.92	pCNP-SLB
Carbonyl group (c) (C=O)	1632	42.10	pCNP
	1626	12.62	SLB
	1635	71.46	pCNP-SLB
Inorganic Phosphate (d) (P=O)	1067	10	pCNP
	1081	40.15	pCNP-SLB
Aromatic ring (e) (C=C)	1507 & 1461	38.53 & 30.42	SLB
	1536 & 1465	81.63 & 41.83	pCNP-SLB
Ether (f) (C–O–C)	1268	10	SLB
	1223	67.95	pCNP-SLB

The characteristic functional groups are labeled for (**a**) amine group, (**b**) alkyl group, (**c**) carbonyl group, (**d**) inorganic phosphate group, (**e**) aromatic ring and (**f**) ether group.
